# Investigating the spatiotemporal characteristics and medical response during the initial COVID-19 epidemic in six Chinese cities

**DOI:** 10.1038/s41598-024-56077-3

**Published:** 2024-03-25

**Authors:** Li Lan, Gang Li, Muhammad Sajid Mehmood, Tingting Xu, Wei Wang, Qifan Nie

**Affiliations:** 1 College of Urban and Environmental Sciences, Northwest University, Xi’an, 710127 China; 2 Shaanxi Key Laboratory of Earth Surface System and Environmental Carrying Capacity, Northwest University, Xi’an, 710127 China; 3 College of Geography and Environmental Science, Henan University, Kaifeng, 475001 China; 4 Natural Resources Bureau of Shuocheng District, Shuozhou, 036000 Shanxi China; 5 Alabama Transportation Institute, The University of Alabama, Tuscaloosa, AL 35487-0288 USA

**Keywords:** Viral infection, Environmental social sciences

## Abstract

In the future, novel and highly pathogenic viruses may re-emerge, leading to a surge in healthcare demand. It is essential for urban epidemic control to investigate different cities’ spatiotemporal spread characteristics and medical carrying capacity during the early stages of COVID-19. This study employed textual analysis, mathematical statistics, and spatial analysis methods to examine the situation in six highly affected Chinese cities. The findings reveal that these cities experienced three phases during the initial outbreak of COVID-19: “unknown-origin incubation”, “Wuhan-related outbreak”, and “local exposure outbreak”. Cities with a high number of confirmed cases exhibited a multicore pattern, while those with fewer cases displayed a single-core pattern. The cores were distributed hierarchically in the central built-up areas of cities’ economic, political, or transportation centers. The radii of these cores shrank as the central built-up area’s level decreased, indicating a hierarchical decay and a core–edge structure. It suggests that decentralized built environments (non-clustered economies and populations) are less likely to facilitate large-scale epidemic clusters. Additionally, the deployment of designated hospitals in these cities was consistent with the spatial distribution of the epidemic; however, their carrying capacity requires urgent improvement. Ultimately, the essence of prevention and control is the governance of human activities and the efficient management of limited resources about individuals, places, and materials through leveraging IT and GIS technologies to address supply–demand contradictions.

## Introduction

The coronavirus disease 2019 (COVID-19) is an infectious disease caused by a novel strain of the coronavirus family, namely severe acute respiratory syndrome coronavirus 2 (SARS-CoV-2)^1^, which belongs to the same viral family as SARS-CoV-1 responsible for the global outbreak of severe acute respiratory syndrome (SARS) in 2003^2^. As of August 26, 2022, over 596 million confirmed cases of COVID-19 have been reported worldwide to the World Health Organization (WHO). Despite efforts to control its spread, the COVID-19 pandemic persists in numerous countries due to highly transmissible variants such as Delta and Omicron^3,4^. This ongoing crisis has profoundly disrupted societal norms and inflicted significant economic repercussions on a global scale. Consequently, containment and mitigation strategies have emerged as the prevailing focus in fields such as medicine, health, public administration, and city planning.

In retrospect, there have emerged highly virulent viruses such as cholera, typhus, smallpox, measles, tuberculosis, leprosy, and malaria, alongside grave epidemics like the Black Death (1331–1353), the Great Plague of London (1665–1666), the San Francisco plague (1900–1904) and the Spanish flu (1918–1920). Over the past two decades alone, Africa, Asia, Europe, and Arabia have been predominantly affected by outbreaks, including SARS in 2002, Middle East respiratory syndrome (MERS) in 2014, influenza A(H1N1) in 2009, Ebola in 2014 and Zika in 2016^5^. It is worth noting that all these viruses and plagues initially appeared and proliferated within urban areas. Currently accounting for approximately 95% of COVID-19 pandemic cases concentrated in cities^6^, as Alirol et al.^7^ highlighted, metropolitan areas have evolved into epicenters for infectious diseases. Considering that urban populations currently constitute around 55% of the global population with an expected increase to 68% by 2050^8^, it is crucial to comprehend the transmission characteristics of COVID-19 during its early stages within cities to effectively manage future epidemics while mitigating potential public health crises, economic downturns, political tensions, and various social issues.

The transmission of the novel coronavirus is primarily through human contact, and thus, the spread of the COVID-19 pandemic has close links with human activities^9–11^. For instance, in Daegu City in South Korea, “Patient 31” continued to visit public places and attend gatherings such as religious services despite exhibiting symptoms without being diagnosed, resulting in a widespread outbreak within South Korea^12^. The activity patterns shown by the population in a city largely influence the severity and spatiotemporal distribution of urban epidemics. Therefore, analyzing the sociological characteristics of diagnosed patients, spatiotemporal patterns of the outbreak, and the social impact has become increasingly prevalent in epidemiology, public health, and geography over the past few decades^13,14^. The primary objective of such analysis is to identify disease clusters, interpret their spatial distribution patterns and causes, and predict the risk of disease transmission^15,16^. Timely and accurate surveillance of spatial and temporal disease dynamics is crucial for detecting outbreaks and identifying high-risk areas for transmission^17^. Given that infectious disease transmission risk varies across time and space, monitoring spatiotemporal trends in disease occurrence can reveal dynamic patterns of risk and aid in mitigating the spread of diseases. Furthermore, during outbreaks, many cities face overwhelming pressure on medical facilities^18,19^ while also experiencing disparities in their ability to respond to surges in healthcare demands^20^. Therefore, comprehending the early-stage spatiotemporal spread characteristics of COVID-19 and assessing medical carrying capacity can contribute to developing effective measures for policymakers’ response to future novel pandemics.

As China was the first country to report and respond to the COVID-19 outbreak, this study selected six high-prevalence Chinese cities and utilized textual analysis, mathematical statistics, and spatial analysis to compare sociodemographic characteristics of confirmed patients, spatiotemporal distribution of the epidemic, and spatial layout and carrying capacity of medical care in these cities to identify commonalities. The results were subsequently deliberated from the perspective of urban planning, architectural design, and healthcare services, culminating in recommendations for future epidemic prevention and control. These findings aim to furnish policymakers with valuable insights.

## Results

### Sociodemographic characteristics of COVID‑19 confirmed in six Chinese cities

#### Demographic characteristics

**Figure 1:**
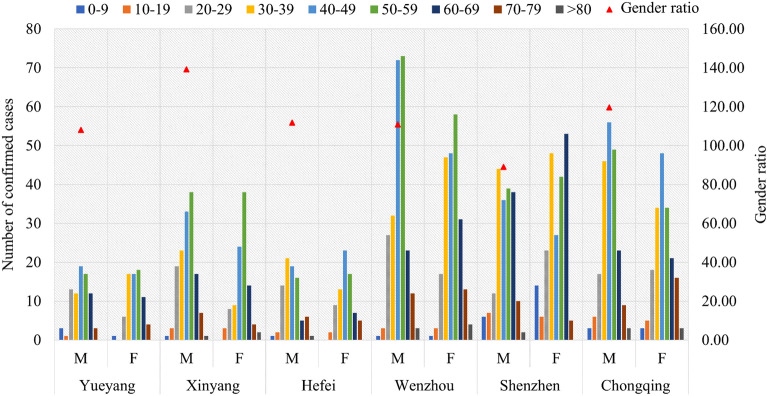
Gender and age distribution of confirmed cases in six Chinese cities. *M* male, *F* female.

The confirmed patients were categorized into nine age groups with 10-year intervals. Figure 1 illustrates that, excluding Shenzhen, the diagnosed patients in the case cities were predominantly male, with gender ratios above 100 and an age concentration between 30 and 59 years. Among them, Xinyang City exhibited the highest gender ratio (139.22), with male confirmed cases between 20 and 49. Conversely, Shenzhen (population gender ratio in 2019 was 101.1) had the lowest gender ratio of confirmed cases (89.04). In Shenzhen, female confirmed cases were concentrated between the ages of 30 and 39 and between 60 and 69 years old. Textual analysis revealed that these findings are associated with activity patterns among confirmed cases: young and middle-aged diagnosed men in Xinyang primarily consisted of migrant workers returning from Wuhan, whereas in Shenzhen, most confirmed cases involved individuals traveling back and forth from other cities.

Wuhan serves as Hubei Province’s capital city and possesses a higher economic status than Xinyang—a prefecture-level city located northwards—thus attracting significant population mobility between both cities due to its greater allure for residents from Xinyang seeking better opportunities. On the other hand, Shenzhen has transformed from a small fishing village that houses merely thirty thousand people into an international metropolis that accommodates millions. This development has benefited from China’s first special economic zone initiative and reform policies during its opening-up period. As a result, Shenzhen has become an attractive destination for numerous young talents who choose to work or reside there. The initial stages of the epidemic coincided with China’s distinctive Spring Festival travel rush, leading to a notable trend of middle-aged and elderly individuals visiting relatives between Shenzhen and other regions. These observations suggest that the early spread of the epidemic is associated with urban economy, culture^21^, and corresponding population movement^22,23^.

#### Trajectory characteristics of confirmed cases

**Figure 2:**
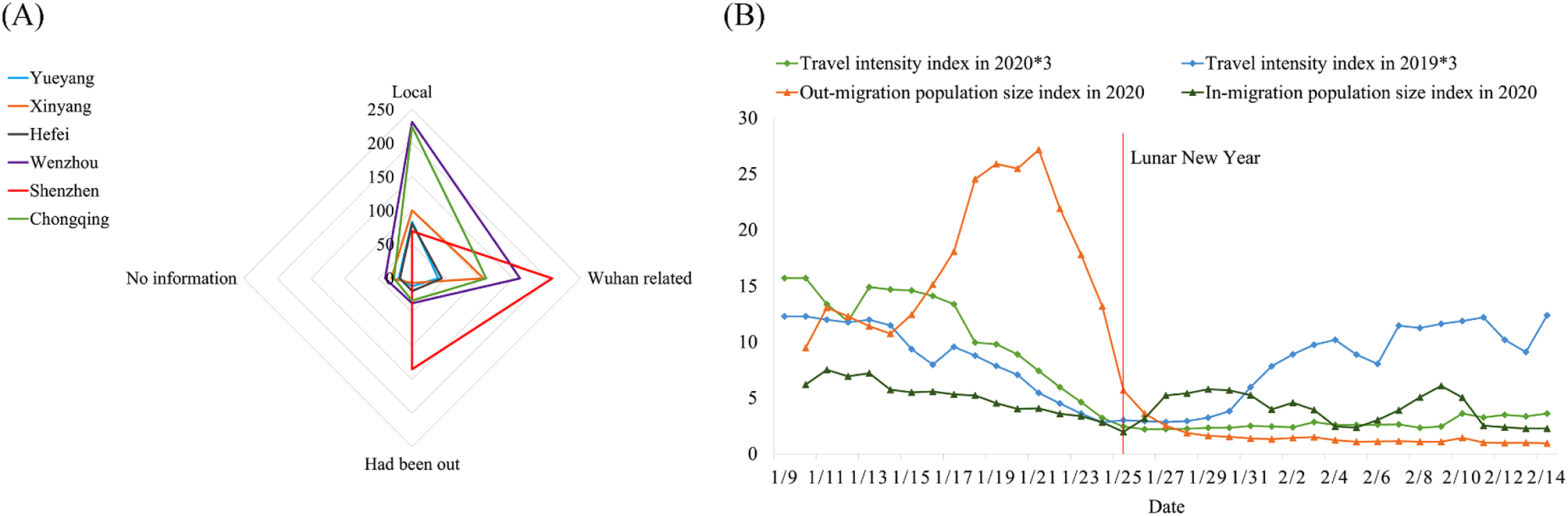
Activity range of confirmed cases in six Chinese cities (**A**) and demographic travel characteristics in Shenzhen, China (**B**).

Trajectory characteristics of confirmed cases were analyzed and categorized based on their activity range, including “Local” (cases with no travel history outside the city), “Wuhan-related” (cases who had visited or transited through Wuhan), “Had been out” (cases who had traveled outside the city without any link to Wuhan), and “No information” (Fig. 2A). Except for Shenzhen, all cities exhibited similar patterns. The activities of confirmed cases in the five cities were primarily local, whereas, in Shenzhen, the activities of confirmed cases were predominantly associated with Wuhan or travel outside the city (most to Hubei Province). Textual analysis revealed that a significant proportion of confirmed cases in Shenzhen consisted of middle-aged and elderly individuals from Wuhan who visited their relatives during the Spring Festival, reflecting both demographic factors (dominance of outsiders) and China’s social culture (reuniting with family during the Spring Festival).

However, the absence of significant local transmission in Shenzhen, a city with a population of tens of millions, despite the presence of numerous confirmed cases related to Wuhan and individuals who had traveled outside the city, can be attributed to Shenzhen’s extensive experience in prevention and control measures accumulated since SARS outbreak and its unique demographic characteristics (Fig. 2B). In 2020, Shenzhen witnessed a sharp increase in its out-migration population size index starting from January 16 while intra-city travel intensity began to decline. Both indices reached their lowest point at the beginning of the Chinese Lunar New Year. The implementation of effective pandemic prevention policies successfully curtailed the growth of both the in-migration population size index and intra-city travel intensity index after the Spring Festival. These factors collectively contributed to impeding the large-scale local spread of the outbreak^24^.

Therefore, it can be inferred that cities’ social, economic, and cultural contexts^21^ as well as population mobility^22,23^ play crucial roles in determining the extent of COVID-19 transmission within cities.

### Spatiotemporal characteristics of the COVID-19 pandemic

#### Temporal evolution process

**Figure 3:**
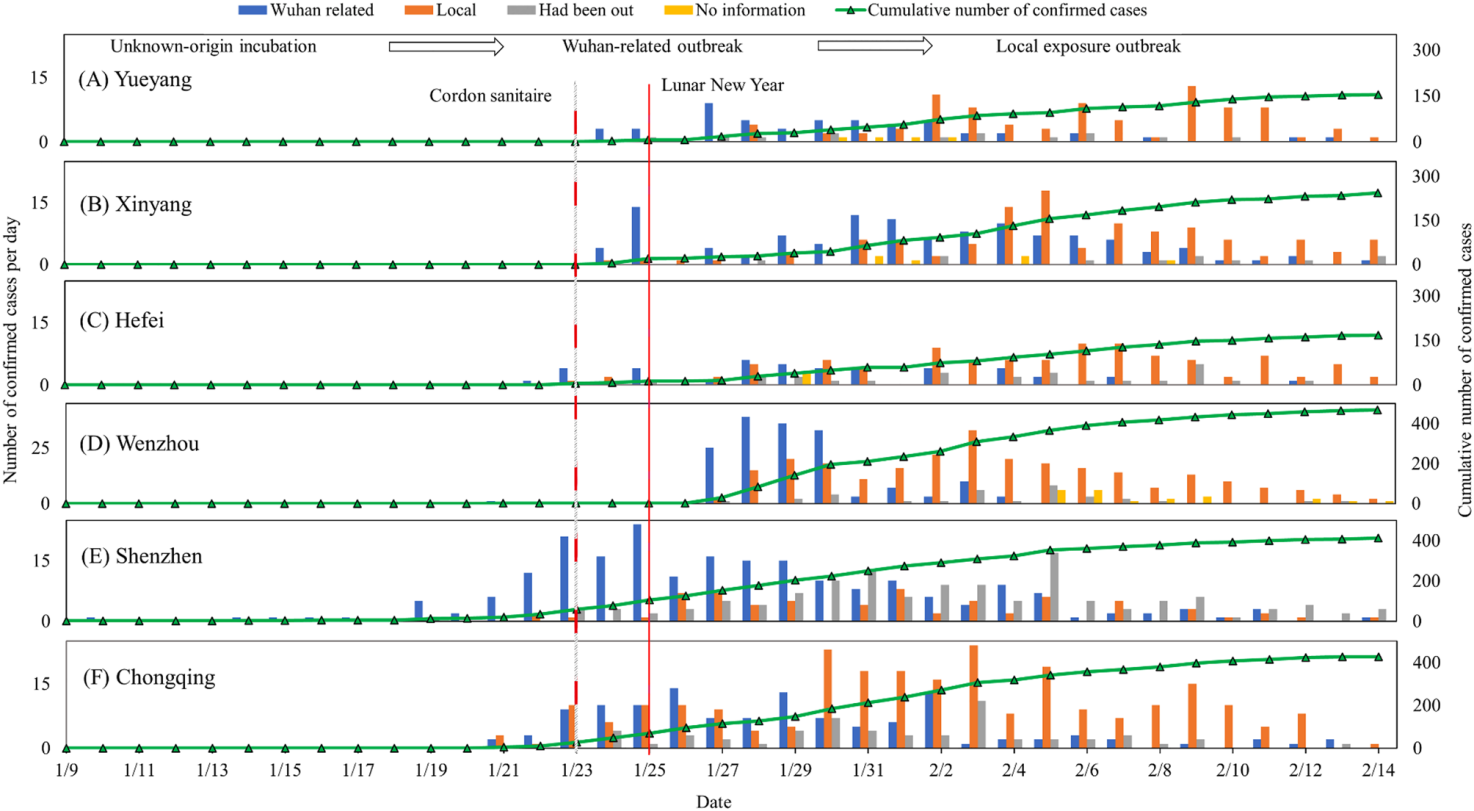
Temporal dynamics of COVID-19 infection in six Chinese cities during 2020.

Based on the classification of COVID-19 confirmed cases by activity range, we depicted the temporal evolution of each category in six cities (Fig. 3). Regarding the daily series of confirmed COVID-19 cases in these cities, the number of “Wuhan-related” cases (blue columns) reached its peak at the end of January, followed by a surge in “Local” cases (orange columns) at the beginning of February. Despite Wuhan’s lockdown measures implemented on January 23, 2020, the incubation period of SARS-CoV-2 and population outflow from Wuhan before travel restrictions resulted in ongoing outbreaks related to Wuhan and subsequent local exposure outbreaks across these six cities. Consequently, the initial spread of COVID-19 within these areas underwent three stages: an unknown-origin incubation period, followed by a Wuhan-related outbreak, and ultimately transitioning into a local exposure outbreak. Furthermore, it is noteworthy that “Local” confirmed cases generally had later dates compared to those classified as “Wuhan-related”, supporting that SARS-CoV-2 exhibits an incubation period and necessitates vigilance for identifying asymptomatic infected individuals^25^.

The cumulative number of confirmed cases (blue lines) increased in late January 2020. Still, it reached a plateau and maintained a stable trend in mid-February due to the implementation of intra-city travel restrictions in six cities. This finding supports the notion that travel restrictions can effectively mitigate the spread of the outbreak^26,27^. However, it is essential to acknowledge that humans have an inherent inclination for unrestricted movement and may find “home confinement” challenging. Therefore, effective pandemic control lies in the rational planning and management of human behavior while simultaneously addressing their basic needs.

#### Spatial distribution patterns

**Figure 4:**
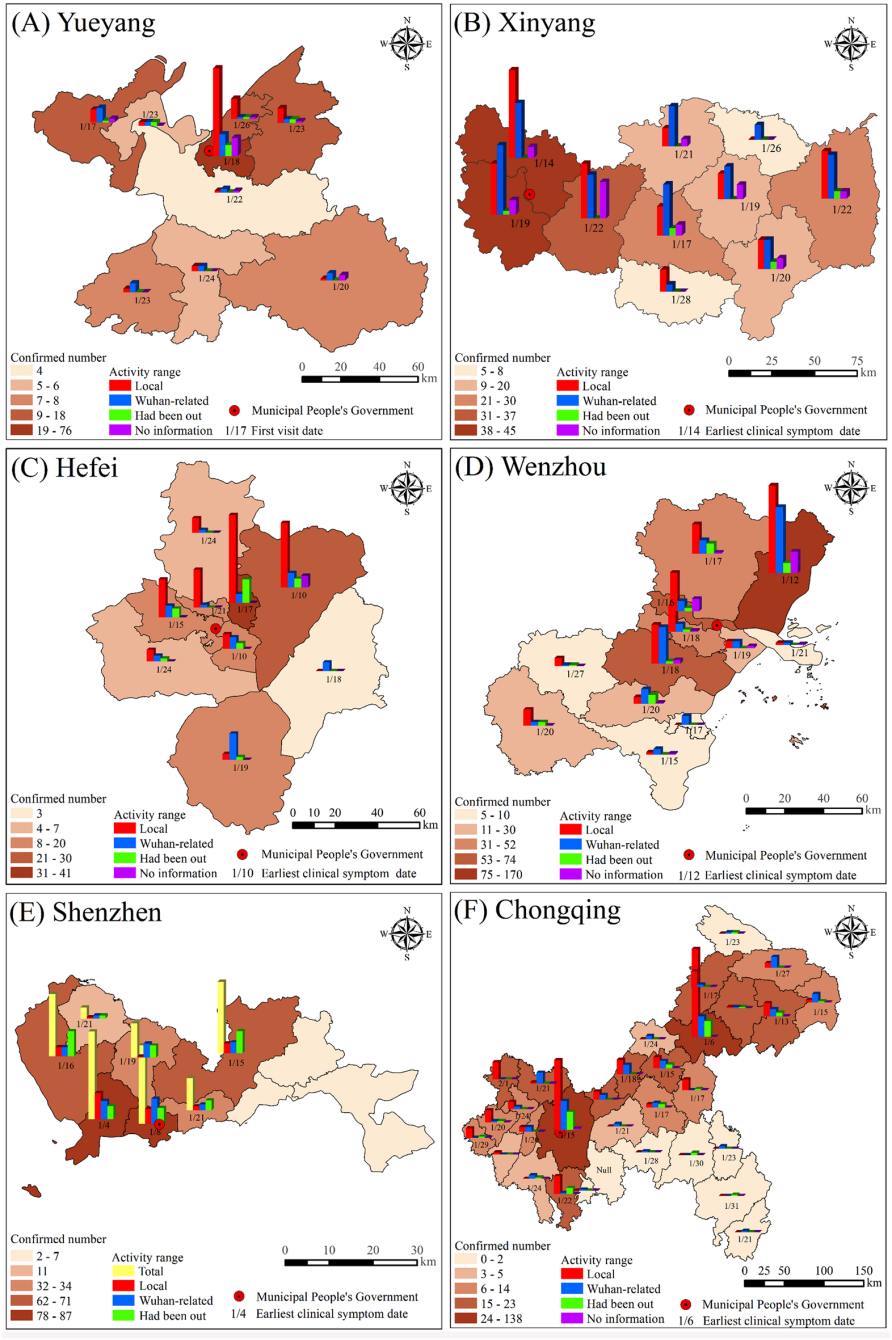
The spatiotemporal distributions of the COVID-19 epidemic in six Chinese cities. In figure (**A**) Yueyang, the first visit date replaced the earliest date of clinical symptoms as it was unavailable. Shenzhen did not provide the “No information” item. This graph was produced using ArcGIS 10.6. https://www.esri.com; the base map is from China Standard Map Service. http://bzdt.ch.mnr.gov.cn/.

By spatially mapping the epidemic distribution across six cities at the district or county level and overlaying the earliest date of clinical symptoms along with the classification of activity range for confirmed cases (Fig. 4), we observed that Shenzhen, China’s special economic zone and international metropolis, exhibited the earliest onset of clinical symptoms on January 4. Then, Chongqing, one of China’s municipalities directly under central government administration and an economically robust city, experienced its first clinical case on January 6. In contrast, Yueyang, a prefecture-level city in Hunan Province (Table 2), had the latest appearance of clinical symptoms on January 17. These findings suggest a positive correlation between earlier symptom onset and factors such as cities’ more developed economies, stronger external connections, and higher levels of administrative authority. However, it is essential to note that early symptom onset does not necessarily indicate a more severe epidemic within a particular city. The severity also depends on other factors, including a city’s history and culture, population mobility patterns, aggregation tendencies, and experience in epidemic prevention and control measures. For instance, despite Shenzhen having the earliest clinical case, its prior experience in SARS prevention and control, effective high-level management, and unique characteristics of population mobility successfully mitigated widespread local transmission (as previously mentioned).

Additionally, there was a more substantial outbreak (indicated by darker colors in Fig. 4) and more “Local” cases in districts or counties with earlier onset of clinical symptoms within the six cities. It highlights the importance of early detection of asymptomatic infections and contact tracing for effective outbreak prevention and control^28^. Moreover, based on the findings presented in Fig. 4, it can be observed that the regions prone to epidemics within these cities (excluding Hefei and Wenzhou) are predominantly situated in districts where the municipal government is located.

**Figure 5:**
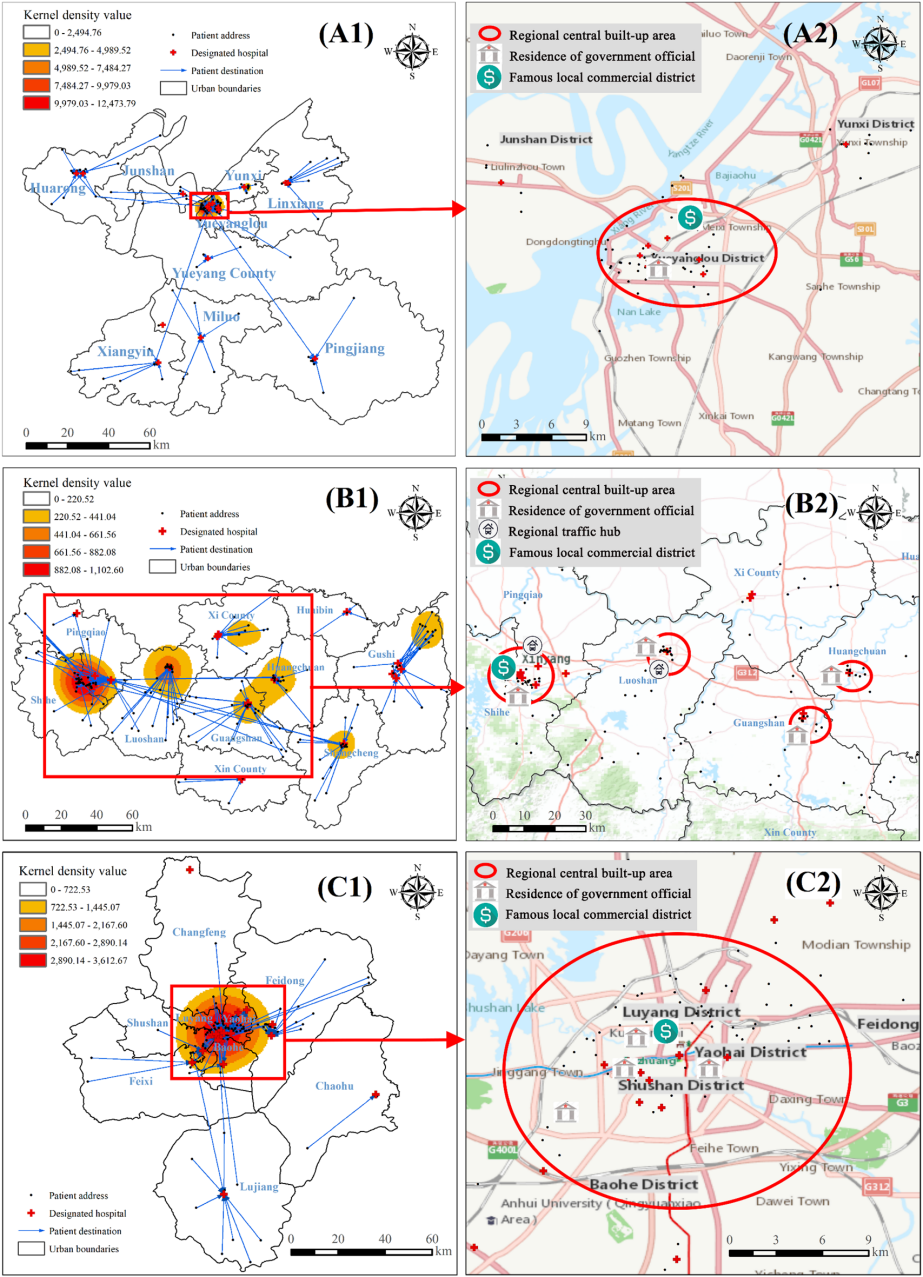
Comprehensive map of urban epidemic and human environment in cities near Wuhan, China. (**A1**,**A2**) Yueyang; (**B1**,**B2**) Xinyang; (**C1**,**C2**) Hefei. Conduct KDE analysis based on confirmed cases’ addresses and overlay this information with a map layer depicting their treatment hospitals to generate figures (**A1**,**B1**,**C1**). Then, select high kernel density value areas from (**A1**,**B1**,**C1**) and overlay them with latitude and longitude data containing the residence of government officials, prominent local commercial districts, and important transportation hubs for symbolic representation. Furthermore, incorporate online base maps from ArcGIS 10.6 to annotate central urban areas to create figures (**A2**,**B2**,**C2**). This graph was produced using ArcGIS 10.6. https://www.esri.com; the base map is from China Standard Map Service. http://bzdt.ch.mnr.gov.cn/.

**Figure 6:**
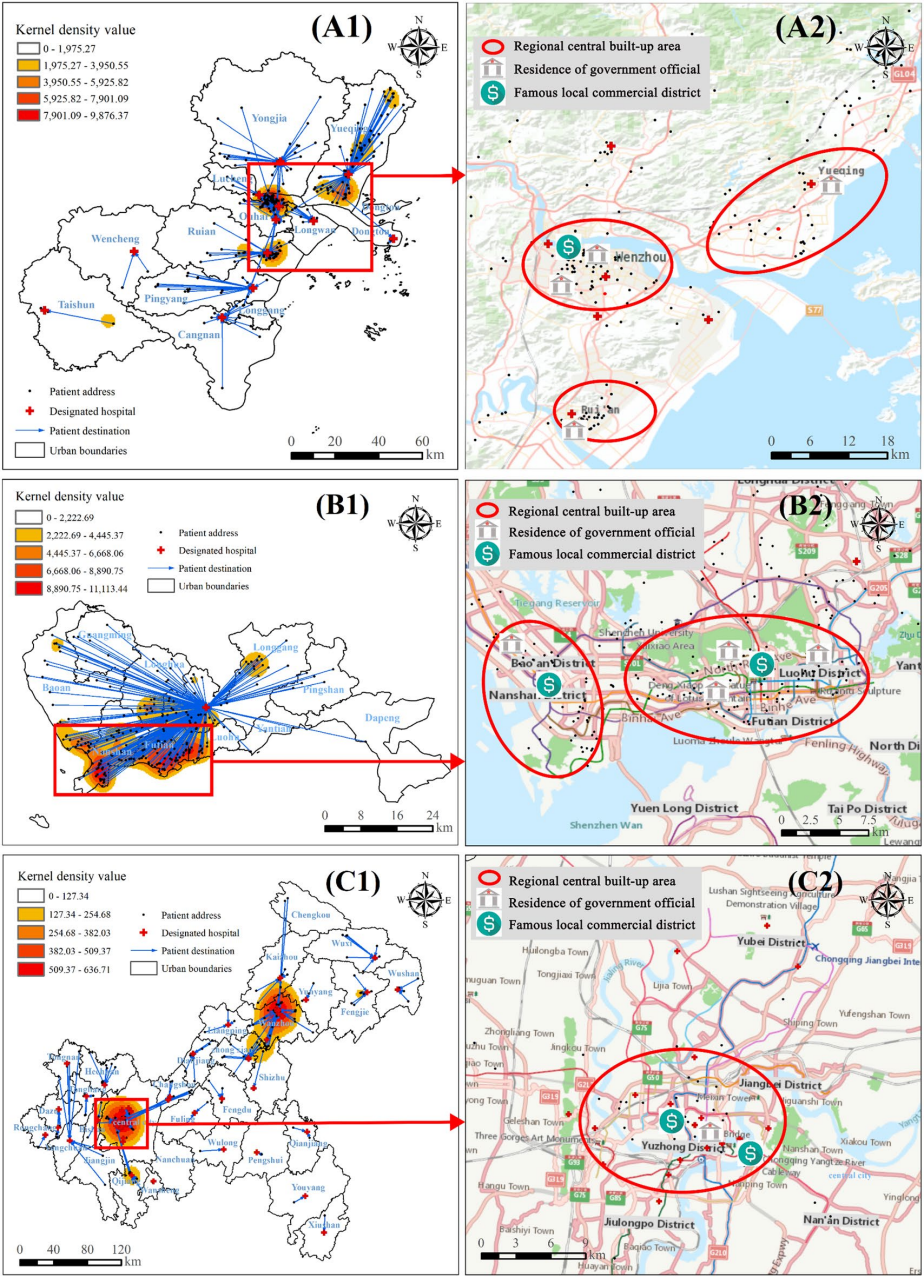
Comprehensive map of urban epidemic and human environment in cities outside Wuhan, China. (**A1**,**A2**) Wenzhou; (**B1**,**B2**) Shenzhen; (**C1**,**C2**) Chongqing. The drawing process is the same as in Fig. 5. This graph was produced using ArcGIS 10.6. https://www.esri.com; the base map is from China Standard Map Service. http://bzdt.ch.mnr.gov.cn/.

By conducting further kernel density estimation (KDE) analysis and overlaying data on residence locations of government officials, prominent commercial districts, important transportation hubs, as well as regional online maps (Figs. 5, 6), we found that clusters of the pandemic did not concentrate on parks, which aligns with the findings reported by Tribby et al.^29^. Instead, they tended to occur within central built-up regions hosting political, economic, or transportation centers such as Yueyanglou District in Yueyang (Fig. 5A1,A2), Nanshan and Futian Districts in Shenzhen (Fig. 6B1,B2), Chongqing’s main urban area (Fig. 6C1,C2), as well as Luoshan county serving as a crucial transportation hub connecting Xinyang with other cities (Fig. 5B1,B2). Notably, convergence points between two or more administrative areas characterized by superior natural and social attributes often attract diverse individuals and entities, forming significant pandemic hotspots. Notable examples encompass the junction between Shihe and Pingqiao Districts (Fig. 5B1), as well as the primary urban areas in both Hefei (Fig. 5C1) and Wenzhou (Fig. 6A1); these regions also serve as the political and economic hubs of the respective cities.

Furthermore, we observed that within the city, the pandemic diminished as the central built-up area’s level decreased, which can be illustrated by the different sizes and color shades of the KDE kernels. For example, in Xinyang (Fig. 5B1), the most severe outbreak occurred at the intersection of Pingqiao District (the municipal government station) and Shihe District (the commercial and financial center in Xinyang), characterized by the largest and darkest KDE core. The sub-serious pandemic emerged within Luoshan County’s built-up area (a vital transit hub for Xinyang’s external connections), while less severe outbreaks existed in other counties’ built-up areas such as Huangchuan County and Guangshan County, with relatively lower political or economic levels, indicated by smaller radii and lighter colors of KDE cores.

Besides, the areas with the highest number of confirmed cases in urban administrative units typically exhibited the highest KDE values. However, Wenzhou presented an exception as its main metropolitan area had the highest KDE value while its county-level city, Yueqing (Fig. 4D), reported the highest number of confirmed cases along with three smaller and dispersed outbreak cores (Fig. 6A). This discrepancy can be attributed to Yueqing’s broader jurisdictional area and more scattered built-up environment compared to the main urban area, resulting in a less concentrated distribution of the pandemic. Consequently, it can be inferred that decentralized built environments pose a lower risk for large outbreaks when compared to concentrated built environments found in political and economic centers.

From the overall epidemic distribution of six case cities in China, we observed that cities with a high number of confirmed cases typically exhibited a multicore pattern, whereas those with fewer cases displayed a single-core pattern. Specifically, Yueyang and Hefei had relatively low confirmed cases (Table 2), demonstrating the single-core pattern (Figs. 5 and 6). In contrast, the remaining cities experienced a significant number of confirmed cases, showing a multicore pattern. Among them, Xinyang is under the jurisdiction of Henan Province but exhibits more robust population mobility (as described above) with Wuhan due to Wuhan’s higher economic level and geographical proximity, resulting in numerous confirmed cases and a multicore pattern. The cities of Wenzhou (Wuhan is known as Wenzhou businessmen’s “second hometown”) and Shenzhen (known for its thriving economy in China, primarily influenced by outsiders) are geographically distant from Wuhan, but maintain close socioeconomic ties with Wuhan, resulting in a significant influx of population movement, a higher number of confirmed cases and the emergence of a multicore structural pattern.

In conclusion, the initial spatiotemporal spread of the epidemic across six cities in China is associated with regional political, economic, and transportation factors, as well as the characteristics of the built-up environment.

### Medical response to the early COVID-19 pandemic in six Chinese cities

#### Spatial distribution of designated hospitals

Overlaying the layers of government-designated hospitals, confirmed cases, and their assigned hospitals with the layers of KDE, we observed that the spatial distribution of designated hospitals (red crosses in A1, B1 and C1 in Figs. 5 and 6) in case cities (excluding Shenzhen) followed a pattern of “local concentration, overall balance”—more designated hospitals were located in central urban areas while fewer were present in counties (mostly limited to “County People’s Hospital” which typically offered better facilities). Shenzhen exhibited a unique characteristic. It had 49 designated medical institutions^30^, but its confirmed cases were all admitted to No. 3 People’s Hospital (Fig. 6B1) due to the hospital’s more advanced equipment and specialized knowledge in infectious disease treatment and management, as well as the city’s extensive experience gained from SARS control measures and relatively small administrative area (Table 2). Additionally, the majority of the confirmed patients were transferred to designated hospitals within the district or county where they developed symptoms (blue arrows in A1, B1, and C1 in Figs. 5 and 6), with only a few cases being referred to higher-level hospitals.

Moreover, areas with the highest KDE values correspond to multiple designated hospitals, whereas regions with lower values have only one designated hospital. It indicates that the medical deployment in case cities was consistent with the spatial distribution of the epidemic.

#### Medical carrying capacity

According to the Guiding Principles for Medical Institution Setting Planning (2016–2020) issued by the Chinese National Health and Family Planning Commission in 2016, the number of beds in medical and health institutions should meet a standard of six per 1000 permanent residents by 2020. However, less than half of the six cities meet this criterion (Table 1), indicating an insufficient carrying capacity of their medical institutions. Although these cities did not experience overload during the outbreak (with low COVID-19 *MCCI*s), it does not imply their ability to withstand a more severe epidemic. With ongoing urbanization leading to continued population concentration in China’s urban areas, particularly within three major urban agglomerations southeast of the Hu Line^31^, as well as global projections indicating an increase in urban population to 68% by 2050^8^, addressing how to flexibly match concentrated urban populations with limited medical and health resources becomes crucial for building resilient cities capable of dealing with public health emergencies. This issue will be further discussed in the following section.

**Table 1:** Medical carrying capacity of six Chinese cities.

City	Number of medical and sanitary beds in 2018/10,000	*MCCI*/1000	COVID-19 *MCCI*
Yueyang	3.366	5.831	0.005
Xinyang	3.636	5.625	0.007
Hefei	5.220	6.374	0.003
Wenzhou	4.236	4.556	0.012
Shenzhen	5.132	3.819	0.008
Chongqing	23.190	7.422	0.002

## Discussion

### Spatial distribution characteristics of the epidemic

First, our results indicate that in the initial stage, the outbreak concentrations were predominantly in the centrally built-up areas in a region’s economic, political, or transportation centers. These central built-up areas typically encompass diverse city functions and possess well-developed built environments and exceptional services, leading to extensive human mobility, significant population aggregation, and high population density. Consequently, these areas emerged as the most severely affected regions during the COVID-19 epidemic^32–34^. In other words, early in the outbreak, COVID-19 outcomes were typically highest in areas with high population mobilities and densities, and this pattern was evident^35^.

Second, the results indicated that at a macroscopic level within a city, there is a reduction in the severity of the regional epidemic as the level of the central built-up area declines. Additionally, at a microscopic level, neighborhoods farther away from central built-up areas exhibit fewer recorded cases^36^. These findings indicate that during the early stages of the pandemic without intervention, the epidemic spread was not constrained by administrative boundaries and distances but rather influenced by urban planning, layout, and socio-economic-cultural context of a city^21^, which manifested through population mobility^10,22,23^.

To be more specific, structurally, towns within a city always tend to exhibit core-periphery characteristics^37,38^, with the cores representing macroscopic economic, political, or transportation centers or microscopically central built-up areas. The higher the level of administrative functions and the more prosperous the economy of a region, the stronger the attraction of the core becomes, resulting in closer social and economic ties with other regions, a more active and concentrated population, and more severe epidemics^33^. Conversely, periphery or suburban areas generally experience lower epidemic levels^39^ due to their poor built environment density and less prosperous economy, resulting in lower population density, clustering patterns, and mobility. The previous analysis of the Wenzhou epidemic supports this observation.

Besides, within the context of social, economic, and cultural factors, mobility and connectivity also exert a significant influence on the spread of outbreaks, potentially surpassing the impact of population density^40^. For instance, despite the diagnosis of a passenger on the Diamond Princess cruise ship in Japan on February 1, 2020, the vessel underwent a month-long quarantine at Yokohama port, effectively preventing a substantial outbreak within Japan^41^. Conversely, in Daegu City, South Korea, “Patient 31” continued to appear in public gathering places frequently even after experiencing symptoms, resulting in a severe domestic epidemic^12^. Moreover, the assertion is also supported by our previous analysis that Shenzhen’s distinctive foreign population structure and outflow during the Spring Festival impeded the spread of the local epidemic. Therefore, effective regulation of social and economic elements, as well as human activities (especially early-stage symptomatic individuals), can contribute to epidemic control^42^.

In summary, the spread of the epidemic exhibits a hierarchical decay and a core-periphery structure; a decentralized built environment is less likely to create a large-scale epidemic cluster. The COVID-19 pandemic, which has dealt a heavy blow to the world’s society and economy and has yet to subside, has prompted urbanists to rethink urban planning and architectural design^5,43^. Are the current agglomerated urban forms characterized by high-rise buildings livable? How can cities effectively reconcile the tripartite challenge of balancing economic development, epidemic prevention and control, and human well-being during the epidemic?

### Future urban planning and architectural design

During the initial stages of the pandemic, nations worldwide implemented diverse control measures on population activities, including the closure of commercial service facilities and sports and recreational venues, stay-at-home orders, and travel restrictions^44^. These measures resulted in a reduction in outdoor physical activity among urban residents^45^, a decline in social interactions in public spaces, and a deterioration of both physical and mental health levels across many populations and countries^46,47^. Additionally, there was an increase in tele-activities, including teleconferencing, telework, telehealth services, online learning platforms, virtual meetings with friends and family members, online live concerts, and virtual weddings^48^. Following the end of lockdowns, people expressed an urgent need for social interaction within communal areas^49^, as well as a strong desire to reconnect with nature by spending time in green spaces^50^.

These phenomena, arising from the COVID-19 epidemic, emphasize the imperative of reshaping urban development patterns in the post-pandemic era. The future urban social and living spaces are likely to witness significant transformations. In response to the surged demand for green and outdoor activities during the pandemic, cities such as San Francisco and Birmingham^51^ created small pocket parks and converted parking lots into parklets. London established walking paths and safe cycling routes along its main arteries and streets, while New York announced the opening of over 150 km of streets to create safe recreational spaces for socializing. Similar examples can be found in Rome, Mexico City, and other cities^52^. These temporary strategies, while addressing immediate needs and alleviating the pressure of epidemic prevention and control, also reveal the limitations of prevailing agglomerated urban forms characterized by high-rise buildings, dense populations, restricted green outdoor spaces, and small indoor living areas.

Urban planning and architectural design should strive to align with the Sustainable Development Goals, such as sustainable cities and communities and good health and well-being. Furthermore, it is crucial to prioritize people’s welfare by reducing the density of high-rise buildings, expanding outdoor activity spaces, and increasing green spaces rather than focusing more on agglomeration economic effects. Numerous studies have already emphasized the importance of a more equitable distribution of urban land use instead of concentration in specific areas^53^.

Both cities and villages possess their allure, yet the undeniable superiority of urban living conditions exerts a more compelling force, attracting 55% of the global population to reside^8^. Individuals seek an enhanced quality of life, gravitating towards cities due to perceived advantages such as superior employment prospects, higher wages, increased social opportunities, and abundant entertainment options; nevertheless, urban dwellers yearn for the natural splendor and fresh air found in rural areas^54^. Therefore, transcending the dichotomy between urban and rural settings by embracing Howard’s Garden City concept that amalgamates the strengths of both realms to foster novel settlement forms^55^ represents an optimal solution for bolstering community resilience, responding effectively to epidemics, and promoting overall health and well-being.

Besides, in the post-epidemic era, as tele-activities persist, commuting may no longer remain the primary focus for urban residents; instead, there will be an increased demand for spacious living spaces and green spaces. Consequently, physical offline spaces such as corporate offices, retail outlets, and transportation facilities might experience a certain degree of reduction while the minimum required living spaces for families might expand to accommodate home offices, personal gardens^56^, individual balconies, and other residential areas. These changes have the potential to save commuting time, reduce energy consumption, increase daily leisure time, and provide access to nature within one’s home environment—making them functional and desirable even in the absence of an epidemic^57^.

### Urban medical carrying capacity

The medical institutions in the six Chinese cities did not overload during the epidemic; however, this does not imply they could withstand a more severe outbreak. In reality, the COVID-19 pandemic posed significant challenges to healthcare systems globally. Cities with a high epidemic incidence generally faced shortages of medical resources. For example, during the intense outbreak in Wuhan, China, over ten Fangcang shelter hospitals were established by converting exhibition centers and stadiums to accommodate confirmed patients^58^. Similar large “tent” venues were also constructed in other countries. Melbourne, Australia, witnessed the erection of a prefabricated semi-containerized two-story COVID-19 hospital in a car park^59^. London, United Kingdom, created a 500-bed Nightingale Hospital within Excel Exhibition Center in Docklands with an increased capacity to treat approximately 4000 patients^60^. India repurposed spaces like train carriages to serve COVID-19 patients^61^. These examples collectively demonstrate that medical facilities and workers become overwhelmed during outbreaks^18^ due to limited resources, thus emphasizing that finding ways to maximize their utilization is crucial.

Urban medical services during an epidemic are essentially the management, allocation, and efficient utilization of resources pertaining to individuals, places, and materials to address the imbalance between supply and demand^62,63^. The restructuring of built fabric above during the outbreak is an excellent example of using the static “place” resource. As people and materials are movable, their management and utilization would be significantly improved by leveraging big data, cloud computing, geographic information systems (GIS), artificial intelligence (AI), and other technologies. Therefore, it is imperative to establish a medical resource platform based on the dynamic database of medical personnel and supplies. The database should encompass the professional background, skill level, career stage (in-service staff members, interns, or school students), workplace location, and home address of all (potential) doctors and nurses, and information on the name, production date, shelf life duration, storage location and quantity of all available medical products, as well as these medical resources’ utilization rates and consumption patterns within a region. Subsequently, utilizing real-time hospital carrying capacity data alongside resource consumption metrics enables automatic calculation and development of contingency plans for scheduling medical staff members effectively while facilitating replenishment plans for circulating backup medical supplies. Furthermore, optimizing medical equipment’s lifespan, extending medical drugs’ shelf life, and developing reusable, ultra-light, and intelligent masks are crucial steps towards achieving large-scale, long-term storage capabilities for materials, thereby minimizing wastage of resources while mitigating environmental pollution^64^.

Furthermore, flexibility and location independence of medical and therapeutic activities have enhanced with the advancement of technologies mentioned above and the establishment of digital health systems^65^. A prominent example is telehealth service, which may experience a substantial and enduring increase^66–69^. Consequently, it becomes imperative to disseminate fundamental medical knowledge and foster nursing skills (preferably through compulsory public university courses) to better accommodate remote diagnosis, treatment, and home care wards^70^ to address the scarcity of healthcare professionals and hospitals during pandemics^71^.

### Implications for prevention and control

In summary, the initial spread of the COVID-19 epidemic exhibited hierarchical and core–edge structural characteristics within cities, which is closely related to human activities. Urban medical services during epidemics are essentially the supply–demand contradiction among individuals, places, and materials. Therefore, epidemic prevention and control ultimately require effective planning and management of human activities and limited resources^44^.

However, managing diverse individuals with initiatives and different social, economic, and cultural backgrounds poses significant challenges. The same epidemic prevention policies, such as mask usage, travel restrictions, or medical service responses, implemented in countries with different cultural backgrounds, economic levels, or urban environments may yield disparate outcomes^9,19^. For instance, regarding the issue of mask usage in public places during the pandemic, individuals in predominantly individualistic cultures like Germany and the United States often exhibit reluctance to wear masks and even participate in anti-mask protests^72^. In contrast, individuals in primarily collectivist cultures like China usually wear masks voluntarily. Nevertheless, culture, created by people, has subjective plasticity, while viruses and their detrimental impact on human health are objective. Therefore, it is crucial to establish an objective and scientific epidemic prevention culture while enhancing people’s awareness of infectious diseases and their hazards across different cultural backgrounds for effective policy implementation. In other words, transitioning from managing individuals to educating them about self-management represents the optimal approach for epidemic prevention and control.

Furthermore, given the interconnection of all activities involving individuals, encompassing resource management and allocation, it necessitates a focus on the inherent nature of human behavior, adherence to humanitarian principles, prioritization of people-centered approaches^73^, and maximum employment of information technologies (IT) such as big data, the internet of things, and intelligent monitoring/control systems. Consequently, establishing a regional integrated emergency management GIS^74^ is crucial for precise prevention measures and decision support^75^—a component of smart city development that has been proven beneficial for pandemic control^24^. The regional integrated emergency management GIS should include, at a minimum, a database including dynamic medical case geo-information, a platform for space–time planning of outdoor activities, and a medical resource platform (mentioned in the previous section).

Medical cases with spatial attributes, particularly the earliest confirmed cases, can provide invaluable information on potential high-risk areas for timely control of infection sources, interruption of transmission routes, and early warning of new pandemics. Therefore, it is imperative to establish a dynamic geo-information database dedicated explicitly to medical cases. This database should be based on diagnosis and treatment data and incorporate crowdsourced data such as Chinese Ding Xiang Yuan. Crowdsourcing data offers geotagged high-frequency information and alternative insights that enhance the resolution of disease spatiotemporal analysis while fostering public health awareness through active public engagement processes^76,77^.

The construction of a platform for space–time planning of outdoor activities should be based on the dynamic geo-information database of medical cases above, ensuring normal outdoor activities while reducing direct person-to-person contact, which is the primary mode of SARS-CoV-2 transmission^78^ and other infectious diseases. Before the nationwide lockdown in mid-March in Italy, outdoor leisure time was allowable. However, due to inadequate deployment and intervention measures, gardens and parks became gathering places, exacerbating the contagion risk, leading to the closure of public places and stricter outing restrictions^79^. Hence, a platform for space–time planning of outdoor activities is indispensable. This platform should initially map all available outdoor areas for people’s activities in high-risk regions (including unused driveways and parking lots within control areas). Subsequently, utilizing temporal geography methods, maximum safe trip numbers should be designed for each region, with trips categorized and dynamically counted by households daily and hourly, to reasonably plan, arrange, and adjust the activity locations/ranges and periods/frequencies of individuals with outdoor activity requirements. In this way, utmost efforts can be made to fulfill people’s basic demands for outdoor activities in high-risk areas during epidemics, ultimately regulating and promoting their physical and mental well-being^16^.

In addition, the epidemic spreads hierarchically and exhibits a core-fringe structure; the built environment significantly varies across diverse social, economic, and cultural backgrounds and different groups exhibit distinct activity characteristics. Therefore, it is necessary to formulate tailored policies based on specific temporal and spatial conditions. The principles of prioritizing science approaches, implementing hierarchical planning, fostering situational awareness, promoting people-centric strategies, providing individualized treatment options, and embracing eco-friendly measures should be consistently applied throughout the prevention and control process to effectively address people’s needs, gain their trust, and ultimately garner their support.

### Limitations and prospects

Although our research has generated new knowledge, there are certain limitations. Firstly, the data solely relies on officially reported cases and fails to consider underreporting^80^. Underreporting can significantly impact the study of spatial distribution and prevention and control strategies of the epidemic, including vaccine strategies^81^. Despite the research advancements in measuring the underreporting of infectious diseases^82^, this article focuses on the early stage of the COVID-19 outbreak when cities’ epidemic response strategies were not yet fully developed; thus, it was challenging to avoid underreporting, resulting in incomplete coverage of confirmed cases in this study. Besides, some data had to be excluded due to incomplete information. Nevertheless, existing datasets still exhibit relatively high data coverage (Table 2). Therefore, these findings objectively present current facts with a certain degree of reference value.

**Table 2:** General information in six Chinese cities. The high-speed train time was from the official website of China Railway (https://www.12306.cn/index/); The socioeconomic data came from the 2020 Statistical Yearbook of China Economic and Social Big Data Research Platform (https://data.cnki.net/yearBook?type=type&code=A).

City	Shortest high-speed train time to Wuhan/min	Total population at the end of 2019/10,000	2019 GDP/100 m yuan	Urban land area/km^2^	Administrative rank	Sample size	The coverage rate of the sample (%)
Yueyang	50	577.13	3780.41	14,858	prefecture-level	154	100
Xinyang	43	646.39	2758.47	18,916	prefecture-level	246	98
Hefei	98	818.90	9409.40	11,496	Provincial capital	170	100
Wenzhou	377	930.00	6606.10	12,103	prefecture-level	471	94
Shenzhen	276	1343.88	26,927.09	1998	Sub-provincial city	413	100
Chongqing	369	3124.32	23,605.77	82,370	Municipality directly under the Central Government	441	82

Secondly, the study area is small, which may have an unconvincing influence, but the results are consistent with that of other scholars. It has revealed some commonalities among cities at the beginning of the pandemic, which could extend to different cities and the future novel pandemic prevention.

Thirdly, due to substantial variations in confirmed cases and administrative regions, the study employed KDE with different bandwidth settings to depict the spatial distribution patterns of the COVID-19 outbreak in the six cities. These settings present challenges when comparing between different cities. Still, when considering this research primarily focuses on exploring common characteristics of COVID-19 spatial distribution within each city through a case-by-case approach rather than intercity comparisons, they are deemed appropriate.

Lastly, the formula of *MCCI* needs improvement. There are differences in the treatment needs of citizens for different hospital departments. The treatment needs, and demand levels vary among individuals and diseases, exhibiting constant fluctuations. Therefore, relying solely on population size and the number of hospital beds as indicators for static measurement poses challenges in accurately describing the capacity of an urban medical system. The concept of *MCCI* is based on an exaggerated assumption that all city residents simultaneously generate medical treatment needs. While this scenario would not occur in reality, to some extent, the formula can still reflect the level of medical services and their differences between cities, thereby providing value to this study and justifying its adoption. Moreover, noted that the COVID-19 *MCCI* formula does not consider patients other than those with COVID-19, which may underestimate the actual situation and create a relatively optimistic impression.

Despite these limitations, they also provide directions for future research endeavors. As epidemic prevention and control technologies mature along with governance systems, addressing underreporting becomes feasible. Research exploring spatiotemporal distribution characteristics during other periods of the COVID-19 pandemic is more reliable. Future research on comprehensive investigations into spatiotemporal evolution patterns of epidemics and their relationships with urban environments at an individual city level could validate some results of this study. Additionally, cities with similar characteristics can be selected to compare spatiotemporal patterns of the epidemic using the KDE method with identical bandwidths. Lastly, efforts could focus on improving the *MCCI* calculation formula for COVID-19 to support formulating effective epidemic response strategies for urban medical systems.

## Conclusion

This study investigates the sociodemographic characteristics of early confirmed COVID-19 cases, spatiotemporal distribution patterns of the epidemic, and spatial arrangement of designated hospitals and medical carrying capacity in six Chinese cities by employing textual analysis, mathematical statistics, and spatial analysis methods. The results indicate that the severity of urban epidemics during their initial stages is associated with cities’ political, economic, and transportation levels and built-up area environment. Furthermore, we observe a correlation between epidemic spread and social-cultural-economic backgrounds as well as factors like population mobility. The potential imbalance between population size and medical capacity poses challenges in effectively managing large-scale outbreaks. Consequently, urban epidemic prevention and control essentially involve governing human activities within limited resources to achieve supply–demand equilibrium during such crises.

This research provides fundamental insights for cities to address future large-scale epidemics better. These findings prompt scholars to reflect on the interplay among economic and social-cultural factors in constructing an urban cascade network system while urging urban planners to reconsider planning strategies and architectural designs accordingly. Moreover, this study offers policymakers and urban managers valuable knowledge in formulating resource governance measures alongside effective epidemic prevention and control strategies.

## Materials and methods

### Data and study areas

#### Data

The epidemic dataset in the study is based on the daily update of the Municipal Health Commission. As of 0:00 on February 15, 2020 (Beijing time), when the outbreak was under control and in the lasting period^24^, a total of 2029 information on confirmed patients described in Chinese was extracted through Python 3.5. After thorough data cleaning, 1895 valuable records were retained, containing details of gender, age, address, date of earliest clinical symptoms onset, date of first visit to healthcare facilities for treatment purposes, designated treatment hospital details, and activity range of confirmed patients. Demographic travel characteristics specific to Shenzhen were collected manually from the “Baidu Migration” big data visualization platform (https://qianxi.baidu.com/#/). Vector administrative boundaries data were obtained from China’s National Basic Geographic Information Center (http://bzdt.ch.mnr.gov.cn/).

#### Study areas

**Figure 7:**
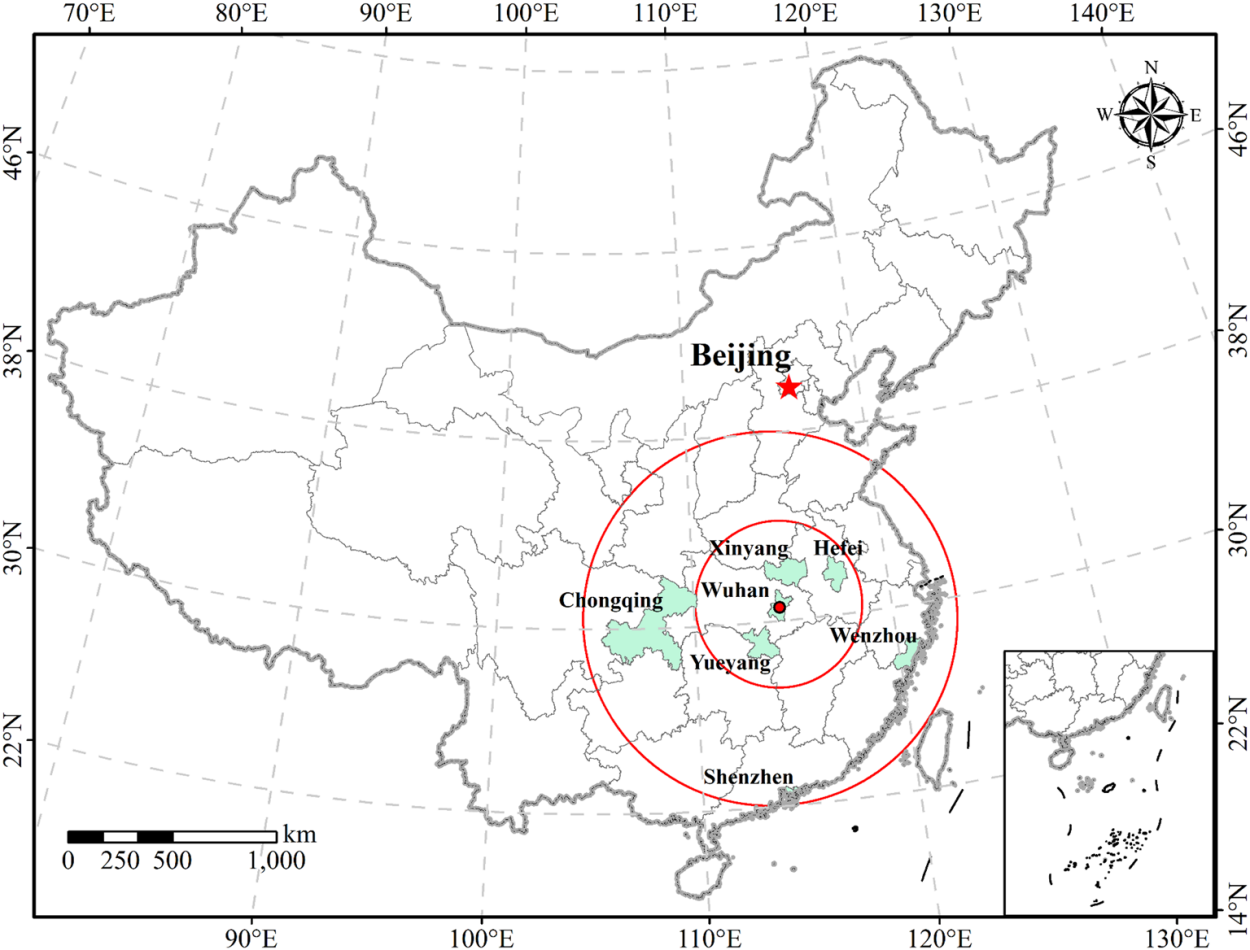
Location of six Chinese cities (this graph was produced using ArcGIS 10.6. https://www.esri.com; the base map is from China Standard Map Service. http://bzdt.ch.mnr.gov.cn/).

The study areas selected for this research include Yueyang, Xinyang, Hefei, Wenzhou, Shenzhen, and Chongqing. Each of these cities possesses distinct geographical, economic, and cultural characteristics. Specifically, the first three cities are in the south, north, and northeast of Wuhan (Fig. 7), all within a two-hour high-speed rail range. On the other hand, the latter three cities are located in the Yangtze River Delta city cluster, Pearl River Delta city cluster, and upper middle Yangtze River region, respectively, requiring more than four hours to reach from Wuhan. These study areas encompass both coastal and inland cities with varying administrative levels, including three prefecture-level cities, one municipality directly under the Central Government, one provincial capital, and one sub-provincial city, with significant variations in city size and economic development level (Table 2). Notably, all these cities experienced severe outbreaks, making them suitable for this research.

### Methods

#### Textual analysis

Textual analysis involves selecting key features from text and quantifying them to represent information. In this study, Python 3.5 was used to extract data such as age, gender, address, and activity trajectory from texts of confirmed cases. The extracted data were then manually cleaned and subjected to statistical analysis. Additionally, manual interpretation and classification of the activity trajectory were conducted, given to rereading the texts when necessary to fully explore their information and assist in explaining the results of statistical analysis.

#### Kernel density estimation

KDE quantifies the spatial distribution of events within a specific area by measuring the density of event points surrounding each location. In this study, we employed KDE to estimate the degree of aggregation for confirmed cases at various locations, aiming to unveil the concentration pattern of the pandemic. The KDE calculation formula is:

1 $${\text{f}}({\text{x}}) = \frac{1}{{{\text{nh}}_{{\text{n}}} }}\sum\limits_{{{\text{i}} = 1}}^{{\text{n}}} {{\text{k}}\left( {\frac{{{\text{x}} - {\text{x}}_{{\text{i}}} }}{{{\text{h}}_{{\text{n}}} }}} \right)}$$

where *n* is the total number of confirmed cases, *h*_*n*_ is the bandwidth, namely the search radius, and ${\text{k}}\left( {\frac{{{\text{x}} - {\text{x}}_{{\text{i}}} }}{{{\text{h}}_{{\text{n}}} }}} \right)$ is the kernel function. Due to significant variations in both the number of confirmed cases and administrative areas among the six cities studied, a uniform bandwidth was not employed. Instead, default settings provided by ArcGIS 10.6 software’s KDE analysis tool were utilized. Specifically, for Yueyang, Xinyang, Hefei, Wenzhou, Shenzhen, and Chongqing, respectively, search radii of 0.057°, 0.191°, 0.045°, 0.081°, 0.039°, and 0.358° were automatically calculated based on spatial configuration and input point numbers without manual adjustments.

#### Spatial overlay analysis

The spatial overlay analysis involves the superimposition of two or more layers of geographic objects within the same area, generating multiple attribute features for that spatial region. It encompasses visual information overlay, vector layer overlay, and raster layer overlay. In this study, only visual information overlay analysis was employed. It entailed utilizing ArcGIS 10.6 to superimpose and visualize multiple layers of information such as designated hospital locations, addresses and treatment hospitals for confirmed cases, kernel density distribution of the outbreak, and regional government office locations as required. This approach was undertaken to investigate their shared characteristics regarding spatial patterns.

#### Medical carrying capacity

Based on the infrastructure capacity index formula^83^ and the guidelines outlined in the “Guideline for Setting Up Medical Institutions (2016–2020)” by the Chinese National Health and Family Planning Commission in 2016, the present study developed a medical carrying capacity index (*MCCI*) that considers the number of permanent residents as the target for carrying capacity, while utilizing the total number of beds in medical and health institutions as an indicator. To adapt it for COVID-19 analysis, we replaced the number of permanent residents with the cumulative number of confirmed patients in *MCCI* while interchanging the numerator and denominator to enhance practicality and comprehensibility. The calculation formulas are as follows:

2 $${\text{MCCI}} = \sum\limits_{{{\text{i}} = 1}}^{{\text{n}}} {{\text{CCM}}_{{\text{i}}} {\text{/CCO}}_{{\text{i}}} \times 1000}$$

3 $${\text{COVID - 19}}\;{\text{MCCI}} = \sum\limits_{{{\text{i}} = 1}}^{{\text{n}}} {{\text{CCA}}_{{\text{i}}} {\text{/CCM}}_{{\text{i}}} }$$

where *n* is the total number of districts or counties under the jurisdiction of case cities; ${\text{CCM}}_{\text{i}}$ are the *i*th district or county medical institution’s total beds; ${\text{CCO}}_{\text{i}}$ is the resident population of the *i*th district or county; ${\text{CCA}}_{\text{i}}$ is the total cumulative diagnoses in the *i*th district and county.

## Data Availability

The epidemic dataset in the study was from the Municipal Health and Wellness Committee websites of the six case cities. Their links are listed as follows: http://wsjkw.xinyang.gov.cn/a/zhuanti/yxhnlgfk/xxgzbd/yqtb/2020/0201/4575.html, https://wsj.yueyang.gov.cn/11161/60061/63074/content_1847025.html/, https://wjw.hefei.gov.cn/public/17771/109495472.html, https://wjw.wenzhou.gov.cn/art/2020/2/11/art_1209919_41899936.html, http://wjw.sz.gov.cn/yqxx/content/post_7343650.html, https://wsjkw.cq.gov.cn/ztzl_242/qlzhxxgzbdfyyqfkgz/yqtb/). The dataset is available from the corresponding author upon reasonable request.
